# Processing of Airborne Green Leaf Volatiles for Their Glycosylation in the Exposed Plants

**DOI:** 10.3389/fpls.2021.721572

**Published:** 2021-11-16

**Authors:** Koichi Sugimoto, Yoko Iijima, Junji Takabayashi, Kenji Matsui

**Affiliations:** ^1^Tsukuba-Plant Innovation Research Center, University of Tsukuba, Tsukuba, Japan; ^2^Department of Applied Chemistry, Kogakuin University, Tokyo, Japan; ^3^Center for Ecological Research, Kyoto University, Kyoto, Japan; ^4^Graduate School of Sciences and Technology for Innovation, Yamaguchi University, Yamaguchi, Japan

**Keywords:** green leaf volatiles (GLVs), glycosylation, bioconversion, specialized metabolism, aldehyde reductase, esterase

## Abstract

Green leaf volatiles (GLVs), the common constituents of herbivore-infested plant volatiles (HIPVs), play an important role in plant defense and function as chemical cues to communicate with other individuals in nature. Reportedly, in addition to endogenous GLVs, the absorbance of airborne GLVs emitted by infested neighboring plants also play a major role in plant defense. For example, the exclusive accumulation of (*Z*)-3-hexenyl vicianoside in the HIPV-exposed tomato plants occurs by the glycosylation of airborne (*Z*)-3-hexenol (Z3HOL); however, it is unclear how plants process the other absorbed GLVs. This study demonstrates that tomato plants dominantly accumulated GLV–glycosides after exposure to green leaf alcohols [Z3HOL, (*E*)-2-hexenol, and *n*-hexanol] using non-targeted LC–MS analysis. Three types of green leaf alcohols were independently glycosylated without isomerization or saturation/desaturation. Airborne green leaf aldehydes and esters were also glycosylated, probably through converting aldehydes and esters into alcohols. Further, we validated these findings in Arabidopsis mutants- (*Z*)-3-hexenal (Z3HAL) reductase (*chr*) mutant that inhibits the conversion of Z3HAL to Z3HOL and the acetyl-CoA:(*Z*)-3-hexen-1-ol acetyltransferase (*chat*) mutant that impairs the conversion of Z3HOL to (*Z*)-3-hexenyl acetate. Exposure of the *chr* and *chat* mutants to Z3HAL accumulated lower and higher amounts of glycosides than their corresponding wild types (Col-0 and L*er*), respectively. These findings suggest that plants process the exogenous GLVs by the reductase(s) and the esterase(s), and a part of the processed GLVs contribute to glycoside accumulation. Overall, the study provides insights into the understanding of the communication of the plants within their ecosystem, which could help develop strategies to protect the crops and maintain a balanced ecosystem.

## Introduction

Plant-derived volatile organic compounds play an important role in the interactions between volatile-emitting plants and herbivores, microorganisms, neighboring plants, and different parts of the damaged plants (Arimura et al., 2009). For example, fruit and flower volatiles attract seed dispersers and pollinators, respectively, whereas the vegetative volatiles of herbivore-infested plants not only attract carnivorous natural enemies of herbivores but also prime the defense response in the neighboring receiver plants (Arimura et al., 2009). Green leaf volatiles (GLVs) are the major constituents of herbivore-infested plant volatiles (HIPVs). Through intensive studies in recent decades, the involvement of GLVs in plant–plant interactions has been widely explored. Some GLVs, such as (*E*)-2-hexenal (E2HAL), (*Z*)-3-hexenal (Z3HAL), and (*Z*)-3-hexenol (Z3HOL) are known to induce the accumulation of jasmonic acid (Engelberth et al., 2004; Hu et al., 2019), the expression of defense genes (Farag et al., 2005; Kishimoto et al., 2005), the biosynthesis of defensive compounds (Kishimoto et al., 2006; Kikuta et al., 2011), the emission of *de novo* synthesized volatiles (Farag and Paré, 2002), and the reemissions of absorbed volatiles (Matsui et al., 2012). In certain cases, GLVs emitted by neighboring herbivore-infested plants can serve as aerial messengers to attract natural enemies of the herbivores. For example, in our previous study, we have shown that airborne Z3HOL (emitted by neighboring infested plants) is absorbed and subsequently converted into (*Z*)-3-hexenyl vicianoside (Z3HexVic), a defensive glycoside against common cutworm larvae (Sugimoto et al., 2014).

**FIGURE 1:**
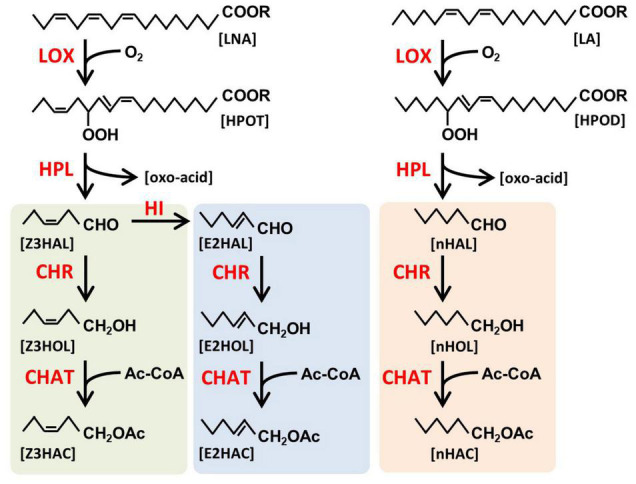
Biosynthetic pathway of green leaf volatiles (GLVs). GLVs are fatty acid-derived volatiles. Linolenic acid (LNA) and linoleic acid (LA) are oxidized by lipoxygenases (LOX) to form 13-hydroperoxy octadecatrienoic acid (HPOT) and 13-hydroperoxy octadecadienoic acid (HPOD), respectively, and subsequently cleaved by hydroperoxide lyase (HPL) to form green leaf aldehydes, (*Z*)-3-hexenal (Z3HAL), and *n*-hexanal (nHAL), respectively. Z3HAL could be converted into (*E*)-2-hexenal (E2HAL) by Z3:E2-hexenal isomerase (HI). Three forms of green leaf aldehydes [Z3HAL (green), E2HAL (blue), and nHAL (orange)] are reduced by cinnamaldehyde and hexenal reductase (CHR) to form green leaf alcohols [(*Z*)-3-hexenol (Z3HOL), (*E*)-2-hexenol (E2HOL), and *n*-hexanol (nHOL), respectively]. The green leaf alcohols are subsequently acylated by acetyl CoA:(*Z*)-3-hexen-1-ol acetyltransferase (CHAT) to form (*Z*)-3-hexenyl acetate (Z3HAC), (*E*)-2-hexenyl acetate (E2HAC), and *n*-hexyl acetate (nHAC) with acetyl CoA as an acyl donor.

Green leaf volatiles have a six-carbon backbone derived from unsaturated fatty acids (Figure 1), and they are categorized into three forms: monounsaturated (*Z*)- and (*E*)-, and saturated *n-*forms (shown as green, blue, and orange background, respectively, in Figure 1). They have three functional groups, aldehyde, alcohol, and esters. It has been shown that the six-carbon Z3HAL, one of the most abundant GLVs from the damaged leaves, and its oxoacid derivative are formed by the cleavage of 13-hydroperoxide (HPOT) by 13-hydroperoxide lyase (HPL, Matsui et al., 1996). HPOT is produced through the oxidization of linolenic acid (LNA) by 13-lipoxygenases (LOX, Halitschke and Baldwin, 2003; Chen et al., 2004). A variety of GLVs are produced by the reduction of aldehyde moiety to form alcohol moiety and subsequent acetylation of alcohol moiety to form esters using the cinnamaldehyde and hexenal reductase (CHR, Tanaka et al., 2018) and acetyl-CoA:(*Z*)-3-hexen-1-ol acetyltransferase (CHAT, D’Auria et al., 2007), respectively. The (*E*)-form of GLVs is formed from its (*Z*)-form by Z3:E2-hexenal isomerase (HI, Kunishima et al., 2016; Spyropoulou et al., 2017). Additionally, when linoleic acid (LA) is processed through this pathway instead of LNA, the saturated *n*-form of GLVs are produced. Some of the GLVs endogenously produced in the leaf tissues accumulate as glycosides, for example, (*Z*)-3-hexenyl primeveroside (Z3HexPri) in the tea leaves (Ohgami et al., 2015; Jing et al., 2019). As evident from these studies, the biosynthetic pathways of endogenous GLVs have been extensively explored; however, little is known about how exogenous airborne GLVs are processed in plants.

In this study, we performed exposure experiments using various GLV species in tomato plants to analyze the metabolic changes in the exposed plants. The expected processing pathways of exogenous GLVs were tested using Arabidopsis mutant plants. The findings of this study could be helpful in the understanding of the communication of the plants within their ecosystem and, therefore, could assist in developing strategies to protect the crops and the natural flora, and maintain a balanced ecosystem.

## Materials and Methods

### Plants and Growth Conditions

Tomato plants (*Solanum lycopersicum*) cv. Micro-Tom were grown on potting soil in a growth room at 25°C with 14 h of fluorescent light (100 μE m^–2^ s^–1^) and for 10 h in the dark. *Arabidopsis thaliana* wild-type Columbia (Col-0), Nossen (No-0), and Landsberg *erecta* (L*er*) were grown on half-strength MS plates in a growth chamber at 22°C with 16 h of fluorescent light (80 μE m^–2^ s^–1^) and 8 h in the dark. Arabidopsis T-DNA insertion mutants of hexenal reductase (*chr*, Tanaka et al., 2018) and acetyl CoA:(*Z*)-3-hexen-1-ol acetyltransferase (*chat*, D’Auria et al., 2007) were developed by the previous work and kindly provided by Prof. J. D’Auria at Texas Tech University, respectively. A pair of the mutant and the corresponding wild-type was grown on a same plate to minimize the environmental effects on their growth and metabolisms.

### Chemicals and Chemical Treatments

Volatile compounds were purchased from Wako Chemicals (Osaka, Japan), except for Z3HAL, which was kindly provided by Zeon Corporation (Tokyo, Japan). Formononetin, an internal standard for liquid chromatography-mass spectrometry (LC–MS) analysis, was purchased from Sigma-Aldrich (St. Louis, MO, United States). For volatile exposure, plants were enclosed in a 2-L glass jar or a 10-L plexiglass box with the volatile-permeated cotton swab at 1 μM (22.4 ppmV) final concentration and exposed for 6 h. Sample leaves were excised from the exposed plants, flash-frozen in liquid nitrogen, and stored at −80°C until use.

### Metabolite Extraction and Liquid Chromatography-Mass Spectrometry Analyses

Metabolite analyses were performed as described by Sugimoto et al. (2014). Briefly, leaf metabolites were extracted by adding 3 × volume of MeOH containing 1 μg mL^–1^ formononetin to ca. 100 mg of ground frozen leaf powder. The extract was injected into the LC-MS (3200 QTRAP, SCIEX, Framingham, MA, United States) or LC-high resolution-mass spectrometry (LC-HRMS; Finnigan LTQ-FT, Thermo Fisher Scientific, Waltham, MA, United States). Chromatographic separation was performed using 0.1% formic acid in water (solvent A) and 0.1% formic acid in acetonitrile (solvent B). The gradient program was started with 20% B for the initial 10 min, 20 to 50% for the next 10 min, followed by an increase from 50 to 95% in 20 min, and a constant 95% B for the final 5 min, with a constant flow rate of 0.2 mL min^–1^ and with an ODS column (Mightysil RP-18, 5 μm, 2 mm × 150 mm, Kanto Chemical, Tokyo, Japan) for LC-MS analysis. The gradient program for LC-HRMS analysis was started with 10% B, followed by an increase to 50% in 50 min, then 90% in 10 min followed by a constant 90% B for the final 5 min with a constant flow rate at 0.5 mL min^–1^, and the separation was achieved with an ODS column (TSK-gel ODS-100V, 5 μm, 4.6 mm × 250 mm, TOSOH, Japan).

### Data Analysis

Pairwise data between mutant and wild-types were compared by *t*-test. Multiple comparisons of the control and volatile treatments were analyzed by Dunnett’s test, wherein, those of the genotype and wounding treatments were analyzed using Tukey’s test. All the statistical analyses were performed using R software (R 4.0.3, R Core Team, 2021) with a threshold of *P* < 0.05. Metabolomic profiles were analyzed using a permutation test with MultiQuant software (SCIEX) with a threshold adjusted at *P* < 0.05.

## Results

### Specific Changes of Tomato Leaf Metabolites by Exposure to Structurally Different Green Leaf Alcohols

**FIGURE 2:**
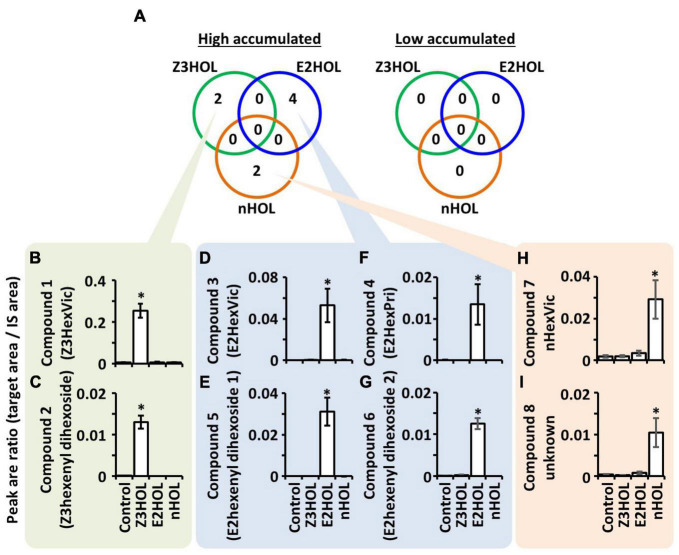
Differentially accumulated compounds in tomato leaves exposed to the green leaf alcohols. **(A)** Numbers in the green, blue, and orange circles represent the differentially accumulated compounds in Z3HOL-, E2HOL-, and nHOL-exposed tomato leaves, respectively. The number of high-accumulated compounds in Z3HOL-, E2HOL-, and nHOL-exposed samples (2, 4, and 2, respectively). No overlapping compounds were found among exposed volatile species. **(B–I)** The relative quantity of each compound is shown in bar graphs (*p < 0.05; Dunnett test). The raw data is available in the Supplementary Data.

Metabolite profiles of tomato leaf extracts were compared with LC-MS when the plants were exposed to the structurally different green leaf alcohols, Z3HOL, (*E*)-2-hexenol (E2HOL), and *n*-hexanol (nHOL). Data processing is summarized in Supplementary Figure 1. Single quadrupole LC-MS analysis detected 2,491 ions in the extracts (Supplementary Data 1). Among 2,491 ions, 19, 15, and 20 ions were high in Z3HOL-, E2HOL-, and nHOL-exposed samples, respectively, compared with the control-exposed samples (Supplementary Data 2). Zero, one, and eight ions were lower in Z3HOL-, E2HOL-, and nHOL-exposed samples, respectively (Supplementary Data 3). The differentially accumulated ions had no overlap among the exposed volatile species. To obtain the rigid dataset, reproducible analysis was performed by LC-HRMS with independent extracts. In Z3HOL-exposed samples, nine ions of the nineteen candidates were reproducibly detected by LC-HRMS (Supplementary Data 4, green colors) and four ions of nine reproduced ones were statistically different from the control-exposed samples (Supplementary Data 4, dark green color). Similarly, nine of the 15 ions were reproducibly detected (Supplementary Data 4, blue colors), and six of the nine reproduced ions were statistically validated (Supplementary Data 4, dark blue color) in high accumulated ions in E2HOL-exposed plants. Seven of 20 in high accumulated ions in nHOL-exposed plants were reproducibly detected (Supplementary Data 4, orange colors) and three of the seven reproduced ions were statistically validated (Supplementary Data 4, dark orange color). On the other hand, no peaks were statistically validated in low accumulated ions even though two ions were reproducibly detected by LC-HRMS (Supplementary Data 5). Finally, four, six, and three reproducible ions were integrated into two, four, and two compounds in Z3HOL-, E2HOL-, and nHOL-exposed samples, respectively (Figure 2, Supplementary Data 6, and Supplementary Figure 1), by considering the retention times (RTs) with chromatographic patterns, isotopic ions, and different adducts as described below.

**FIGURE 3:**
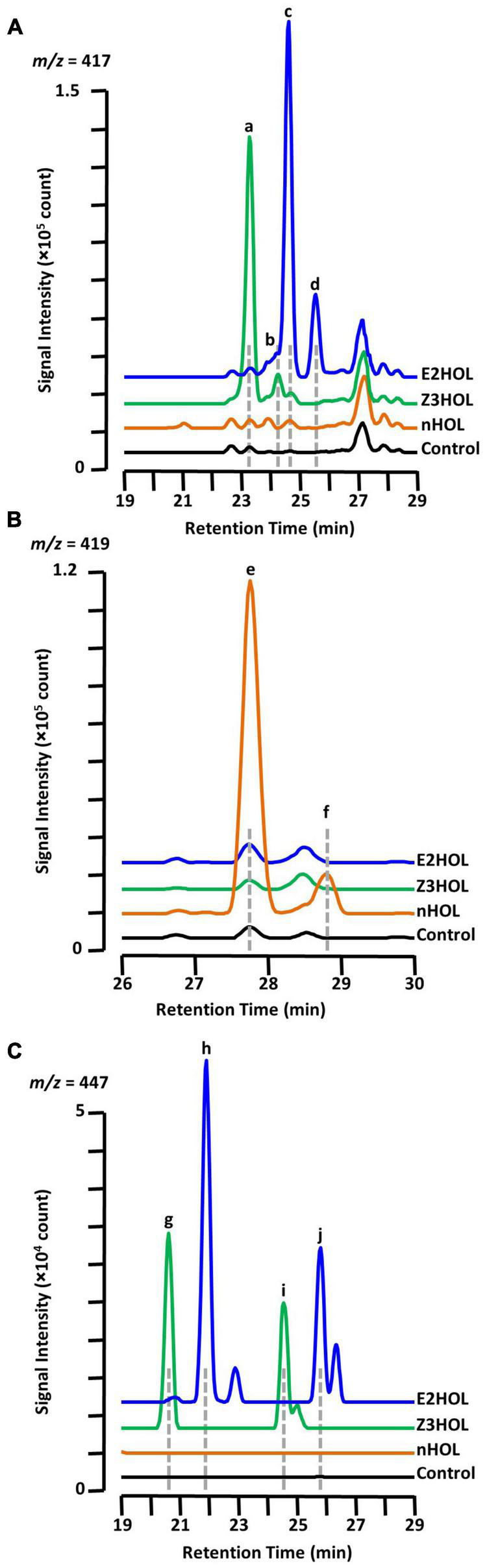
Chromatographic analysis of glycoside accumulation in the green leaf alcohol-exposed tomato. Chromatograms from Z3HOL-, E2HOL-, and nHOL-exposed leaf extracts (green, blue, and orange lines, respectively) were compared at the *m/z* = 417 **(A)**, 419 **(B)**, and 447 **(C)**, corresponding to the green leaf alcohol-diglycoside conjugates. The chromatogram of the control leaf extract is shown by the black line. Peaks a and b are (*Z*)-3-hexenyl vicianoside and (*Z*)-3-hexenyl primeveroside, respectively. Peaks c through f are (*E*)-2-hexenyl vicianoside, (*E*)-2-hexenyl primeveroside, *n*-hexyl vicianoside, and *n*-hexyl primeveroside, respectively. Peaks g and i were (*Z*)-3-hexenyl dihexosides, and peaks h and j are (*E*)-2-hexenyl dihexosides.

It is already known that the Z3HOL-exposed tomato plants accumulate both Z3HexVic (Figure 3A, peak a) and Z3HexPri (Figure 3A, peak b) (Sugimoto et al., 2014). In this analysis, E2HOL-exposed tomato showed a similar chromatographic pattern to Z3HOL-exposed plants with a 1-min delay at *m/z* = 417 (Figure 3A, peaks c and d). Considering the biosynthesis of Z3HexVic and Z3HexPri in Z3HOL-exposed plants, e.g., absorbing and converting the airborne Z3HOL into its glycoside, the peak c and d of Figure 3A in E2HOL-exposed plants putatively derived from the conversion of airborne E2HOL, and were (*E*)-2-hexenyl vicianoside (E2HexVic) and (*E*)-2-hexenyl primeveroside (E2HexPri), respectively (Supplementary Data 6). The similarity of MS and MS/MS spectra of Z3HexVic and E2HexVic gave the additional support to this conclusion. The MS spectrum of Z3HexVic (Figure 3A, peak a) indicated its molecular ion [(M + H)^+^ = 395.19], adduct ions with ammonium [(M + NH_4_)^+^ = 412.22], sodium [(M + Na)^+^ = 417.17] and potassium [(M + K)^+^ = 433.15], and its isotopic ions {[M(^13^C) + Na]^+^ = 418.18, [M(^13^C × 2) + Na]^+^ = 419.18} (Supplementary Figure 2A, MS spectra). The MS spectrum of E2HexVic (Figure 3A, peak c) consisted of the same patterns as Z3HexVic (Supplementary Figure 2B, MS spectra) since the aglycon of two compounds were isomers. The MS/MS fragments of Z3HexVic consisted of a dehydrated (−H_2_O) ion (*m/z* = 377.10), a single sugar-deleted ion (*m/z* = 198.28, Supplementary Figure 2A, MS/MS spectra) with two unassigned ions (*m/z* = 219.08 and 233.09). The MS/MS fragments of E2HexVic consisted of the same assigned ions (Supplementary Figure 2B, MS/MS spectra) with two unassigned ions (*m/z* = 233.17 and 295.01). Under the same concept, nHOL exposure caused similar peak patterns at *m/z* = 419 with a 4.5-min delay from Z3HexVic and Z3HexPri (Figure 3B, peaks e and f). This also suggests that airborne nHOL was likely to be converted into *n*-hexyl vicianoside (nHexVic) and *n*-hexyl primeveroside (nHexPri), reflecting two more hydrogens in nHOL (monoisotopic mass = 102.104) compared with Z3HOL and E2HOL (100.089) (Supplementary Figure 2, structure). The MS spectrum of nHexVic (Figure 3B, peak e) consisted of its molecular ion [(M + H)^+^ = 397.21], adduct ions with ammonium [(M + NH_4_)^+^ = 414.23], sodium [(M + Na)^+^ = 419.19] and potassium [(M + K)^+^ = 435.16], and its isotopic ions {[M(^13^C) + Na]^+^ = 420.19, [M(^13^C × 2) + Na]^+^ = 421.19} (Supplementary Figure 2C, MS spectra). Another ion was detected with high accumulation in nHOL-exposed tomato, which was unassigned by HRMS; therefore the compound remained as an “unknown” compound even with its coelution with nHexVic (Supplementary Data 6).

Under the same context, Z3HOL-exposed plants showed other marginally and significantly accumulated ions with *m/z* = 447 at RT = 20.86 and 24.81 min, respectively (Figure 3C, peaks g and i), which were predicted as hexenyl dihexosides by HRMS. The ions with the same *m/z* were also detected in E2HOL-exposed plants with a 1-min delay in their RTs (Figure 3C, peaks h and j), which was also predicted as hexenyl dihexosides by HRMS. The similar chromatographic patterns between Z3HOL- and E2HOL-exposure implied that these ions were formed by the conversion of airborne volatiles and predicted as (*Z*)-3- or (*E*)-2-hexenyl dihexosides.

### Glycoside Accumulation by Exposure to Different Green Leaf Volatiles

**FIGURE 4:**
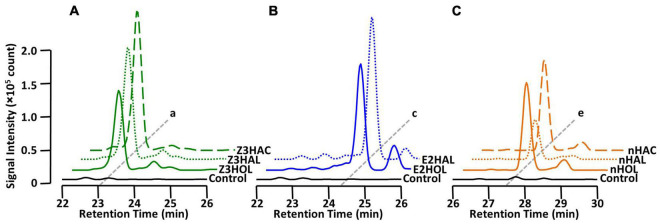
Diglycoside accumulation in GLV-exposed tomato. Comparison of the chromatograms from **(A)** Z3HAL-, Z3HOL-, and Z3HAC-exposed leaf extracts at *m/z* = 417. (*Z*)-3-Hexenyl vicianoside (a) accumulated in the (*Z*)-form GLVs exposed plants. **(B)** E2HAL- and E2HOL-exposed leaf extracts at *m/z* = 417. (*E*)-2-Hexenyl vicianoside (c) was accumulated in both (*E*)-forms of GLV- exposed plants. **(C)** nHAL-, nHOL-, and nHAC-exposed leaf extracts at *m/z* = 419. *n*-Hexyl vicianoside (e) was accumulated in the *n*-form GLVs exposed plants. The chromatogram of the control leaf extract is shown by the black line.

It has been shown that in addition to green leaf alcohols, other groups of GLVs, such as aldehydes and esters, also make the exposed plants defensive (Kishimoto et al., 2005; Frost et al., 2008; Hu et al., 2019; Su et al., 2020). In this study, we tested whether airborne green leaf aldehydes and esters were converted into their corresponding defensive glycosides. The findings demonstrated that exposure of the tomato plants to Z3HAL and (*Z*)-3-hexenyl acetate (Z3HAC) accumulated Z3HexVic similar to Z3HOL exposure (Figure 4A). Similarly, exposure to E2HAL accumulated the same compound found in E2HOL exposed plants, and the exposure of nHAL and *n*-hexyl acetate (nHAC) led to the accumulation of the same compound found in nHOL-exposed plants (Figures 4B,C).

### Estimation of the Z3HexVic Biosynthetic Route From Non-alcohol Green Leaf Volatiles

**FIGURE 5:**
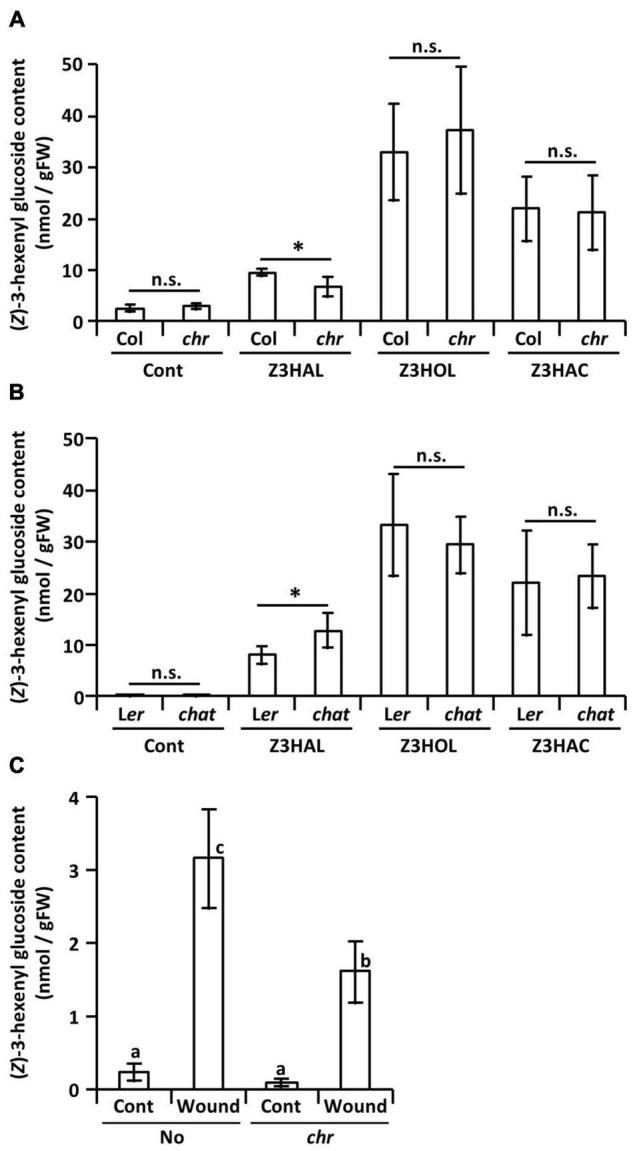
Accumulation of (*Z*)-3-hexenyl glucoside in GLV-exposed and wounded Arabidopsis plants. Comparison of the amount of (*Z*)-3-hexenyl glucoside between **(A)** the *chr* mutant and its corresponding wild type (Col) plants and **(B)** the *chat* mutant and its corresponding wild type (L*er*) plants after (*Z*)-3-GLVs exposure. Wild types and mutants are exposed together in the same plexiglass box as described in Materials and Methods. Data are shown as mean ± SE (*N* = 5–7). The difference between the wild types and mutants was analyzed by *t*-test (*p < 0.05). **(C)** The amount of (*Z*)-3-hexenyl glucoside in the *chr* mutant and its corresponding wild type (No) plants with or without wounding (Wound/Cont) was compared, data are shown as mean ± SE (*N* = 6–20). Different letters indicate the significant difference (*p* < 0.05; Tukey’s multiple comparisons).

Like the endogenous Z3HOL biosynthesis process in plants, the aldehyde moiety of the exogenous Z3HAL is reduced to alcohols. Here, to understand the effect of the mutation on glycoside accumulation, we used a Z3HAL reductase mutant of *Arabidopsis thaliana* (Tanaka et al., 2018). It has been shown that the Col-0 ecotype with a natural mutation in *HPL* gene that cannot produce GLVs (Duan et al., 2005) accumulates (*Z*)-3-hexenyl glucoside (Z3HexGlc) rather than Z3HexVic by Z3HOL exposure (Sugimoto et al., 2015). In addition, we also show that exposure of the Col-0 plants to Z3HAL, Z3HOL, and Z3HAC, accumulated Z3HexGlc (Figure 5A). On the contrary, exposure of the Z3HAL reductase mutant plants (*chr*) to Z3HAL accumulated a lower amount of Z3HexGlc than the Z3HAL-exposed Col-0 plants. However, the *chr* and Col-0 plants accumulated similar levels of Z3HexGlc after Z3HOL and Z3HAC exposure (Figure 5A). It is also known that Z3HOL is esterified by acetyl-CoA:(*Z*)-3-hexen-1-ol acetyltransferase (CHAT) for Z3HAC production. The CHAT mutant (*chat*) and its parental ecotype (L*er*) showed similar levels of Z3HexGlc accumulation following Z3HOL and Z3HAC exposure; however, the *chat* mutant accumulated higher amounts of Z3HexGlc compared with L*er* after Z3HAL exposure (Figure 5B). Additionally, the amount of Z3HexGlc was measured in another *chr* mutant in the No-0 ecotype, which enables the production of endogenous GLVs to test whether the endogenously produced Z3HAL was converted into Z3HexGlc through the Z3HOL biosynthetic pathway. The No-0 plants accumulated Z3HexGlc by wounding treatment; in contrast, the *chr* mutant plants accumulated significantly lower amounts of Z3HexGlc under the wounded conditions (Figure 5C).

## Discussion

### Potential Involvement of the Processing of Volatiles in Defense Induction

Metabolomic analysis of the GLV-exposed tomato leaves indicated that only a limited number of changes occurred, predominantly the glycosylation of exogenous volatiles (Figure 3 and Supplementary Data). Nevertheless, GLV-exposure induces diverse defensive responses against pathogens and herbivores. For example, Arabidopsis plants exposed to Z3HAL and E2HAL elicit a defense against the necrotrophic fungus, *Botrytis cinerea*, with the upregulation of various defense genes (Kishimoto et al., 2005, 2006; Yamauchi et al., 2015). GLV-exposed maize plants show enhanced defensive traits against insect herbivory, *S. littoralis* (Hu et al., 2019) by jasmonate accumulation (Engelberth et al., 2004, 2007; Hu et al., 2019; Gorman et al., 2020), volatile emissions (Farag et al., 2005; Ruther and Kleier, 2005; Yan and Wang, 2006; Hu et al., 2019), and gene expression (Farag et al., 2005; Engelberth et al., 2013; Hu et al., 2019). Z3HAC also elicits an enhanced defense against herbivory in exposed maize plants with primed JA accumulation and gene expression of benzoxazinoid biosynthesis, a specialized defense chemical in maize (Engelberth et al., 2007; Hu et al., 2019). Additionally, Z3HOL exposure in tea plants creates prime conditions for defense gene expression and plant defense against subsequent infestation by the tea geometrid, *Ectropis obliqua* (Xin et al., 2019). Z3HOL exposure also provides tomato plants with increased defense against *Tomato yellow leaf curl virus* transmission by the whitefly, *Bemisia tabaci*, with primed defense gene expression and metabolite accumulation (Su et al., 2020). The large gap between the limited metabolic changes and the diverse defense responses suggest that the processing of GLVs might be involved in defense induction in plants. On the contrary, the substantial accumulation of Z3HexVic begins to be detected ca. 0.5–1 h in the Z3HOL-exposed tomato plants (Sugimoto et al., 2014). This period is much longer than the known “early responses,” such as the change in membrane potential occurring within seconds in the Z3HAL-, E2HAL-, and Z3HAC-exposed tomato plants (Zebelo et al., 2012). Reportedly, MAPK activation was observed around 3 min after the exposure of GLVs in *Lolium* plants (Dombrowski and Martin, 2018), and cytosolic calcium influx was detected at 10 min after E2HAL- and Z3HAC-exposure (Zebelo et al., 2012). Therefore, further studies are required to test whether GLV-glycosylation/-processing is involved in defense induction.

### Processing Pathway of Exogenous Green Leaf Volatiles

**FIGURE 6:**
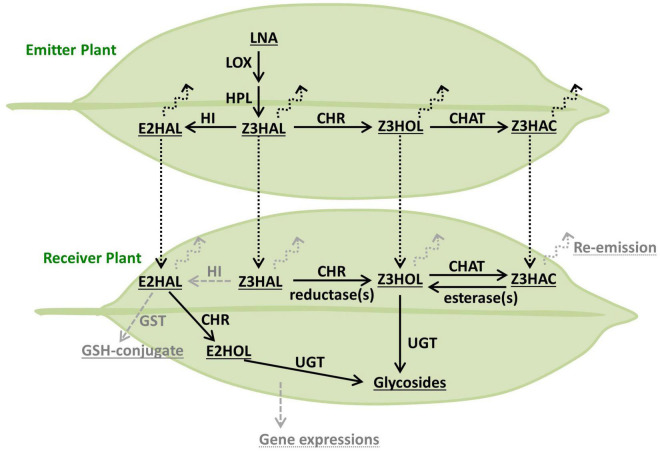
Model of the processing route of green leaf volatiles. The biosynthetic pathway of GLVs originated from LNA is depicted in the emitter plant (top leaf). Processing of exogenous GLVs is depicted in the receiver plant (bottom leaf). The compounds, enzymes, and other processes are described as underlined, plain, and dotted-underlined text, respectively. The enzymatic conversion and airborne transfer are indicated as solid and dotted arrows, respectively. The potential components, which were not found or tested in this study, are shown in gray.

Glycosylation of exogenous Z3HOL is known in various plant species including tomato, Arabidopsis, and tea plants (Sugimoto et al., 2014, 2015; Jing et al., 2019); however, the information on how plants process exogenous green leaf aldehydes and esters is limited. The emission of Z3HOL and Z3HAC by Z3HAL-exposed Arabidopsis plants suggested that the conversion of exogenous aldehyde occurs *via* a pathway similar to that of GLV biosynthesis (Matsui et al., 2012). Here, we showed that the exogenous green leaf aldehydes were converted into their glycosides (Figure 4), and the conversion pathway was partly controlled by the CHR enzyme (Figures 5A, 6), as evident from the mutant analysis. Notably, the findings demonstrated that the *chr* mutant accumulated some Z3HexGlc after exposure to high amounts of Z3HAL or wounding in the Z3HAL producing ecotype. Therefore, it is expected that (an)other enzyme(s) might be involved in the conversion of exogenous Z3HAL into Z3HOL, such as alcohol dehydrogenase (Bate et al., 1998; Speirs et al., 1998).

Interestingly, Z3HAC-exposure accumulated Z3HexGlc in Arabidopsis plants (Figures 4, 5), suggesting the presence of an opposite reaction of GLV biosynthesis mediated by an unidentified enzyme. Z3HAC hydrolysis has also been suggested by Z3HexVic accumulation in Z3HAC-exposed wheat plants (Ameye et al., 2020). The *chat* mutant accumulated significantly higher amounts of Z3HexGlc upon Z3HAL-exposure (Figure 5B), probably because the *chat* mutant impaired the conversion of Z3HOL into Z3HAC, which might cause overaccumulation of Z3HOL, allowing the excess amount of Z3HOL to be used for Z3HexGlc production. This indicates that exogenous Z3HAL is serially converted into Z3HOL and Z3HAC by CHR and CHAT enzymes, respectively. This result supports the previous prediction regarding the re-emission of Z3HOL and Z3HAC from the Z3HAL-exposed plants (Matsui et al., 2012).

### Future Prospects of Green Leaf Volatile-Glycosylation in Plant Defense

Glycosylation of GLVs is a widely deployed defense mechanism in plants (Sugimoto et al., 2014; Jing et al., 2019; Ameye et al., 2020), and the purified Z3HexVic functions as a defensive chemical against herbivory (Sugimoto et al., 2014). Although other defensive glycosides such as hydroxygeranyllinalool diterpene glycosides (HGL-DTGs) accumulated in wild tobacco, *Nicotiana attenuata* (Heiling et al., 2010), have toxic effects (Poreddy et al., 2015), the mode of action of Z3HexVic and other GLV-glycosides remains unknown. Additionally, simultaneous quantitative analysis of the glycosides, the volatiles, and other GLV-derived compounds clarified the complete fate of the exogenous/endogenous GLVs (Figure 6). For example, the absorbed exogenous GLVs are partly reemitted as volatiles and converted to other GLVs (Matsui et al., 2012) and stored as non-volatile compounds such as glycosides (Figures 3, 4; Sugimoto et al., 2014) and glutathione-conjugates (Davoine et al., 2006). To obtain an adequate substrate for glycosylation, absorbed GLVs should be processed through the biosynthetic pathway (Z3HAL to Z3HOL) and its’ reverse pathway (Z3HAC to Z3HOL), depending on the type of airborne GLVs. In this analysis, Z3HAL-exposed tomato plants accumulated an undetectable amount of E2HexVic, even though the functional Z3:E2-hexenal isomerase was present in the tomato genome (Kunishima et al., 2016; Spyropoulou et al., 2017). A potential explanation is that tomato plants might have a low HI activity and therefore emit a reduced amount of (*E*)-2-form GLVs compared with (*Z*)-3-form GLV emission (Degenhardt et al., 2010; Sugimoto et al., 2014). Another possibility is the difference in volatile processing against endogenous GLV production occurring within minutes (D’Auria et al., 2007), and exogenous GLV glycosylation is detectable within hours (Sugimoto et al., 2014). It will also be a future challenge to investigate and understand how exogenous volatiles induce defense responses. Furthermore, the exact mechanism of the absorption/penetration of the airborne GLVs should be explored to clarify the complete mechanism of the defensive roles of GLVs.

## Data Availability Statement

The datasets presented in this study can be found in online repositories. The names of the repository/repositories and accession number(s) can be found in the article/Supplementary Material.

## Author Contributions

KS designed the research and analyzed the data. KS and YI performed the experiments. KS, JT, and KM wrote the manuscript. All authors contributed to the article and approved the submitted version.

## Conflict of Interest

The authors declare that the research was conducted in the absence of any commercial or financial relationships that could be construed as a potential conflict of interest.

## Publisher’s Note

All claims expressed in this article are solely those of the authors and do not necessarily represent those of their affiliated organizations, or those of the publisher, the editors and the reviewers. Any product that may be evaluated in this article, or claim that may be made by its manufacturer, is not guaranteed or endorsed by the publisher.
