# Supplementary material for: Processing of Airborne Green Leaf Volatiles for Their Glycosylation in the Exposed Plants
Source: Front Plant Sci. 2021 Nov 16;12:721572. doi: 10.3389/fpls.2021.721572 (PMC8636985; doi:10.3389/fpls.2021.721572)
Supplement: Supplementary file 1 [file Data_Sheet_1.zip › SupplementaryMaterials/SupplementaryFigure1.pdf]

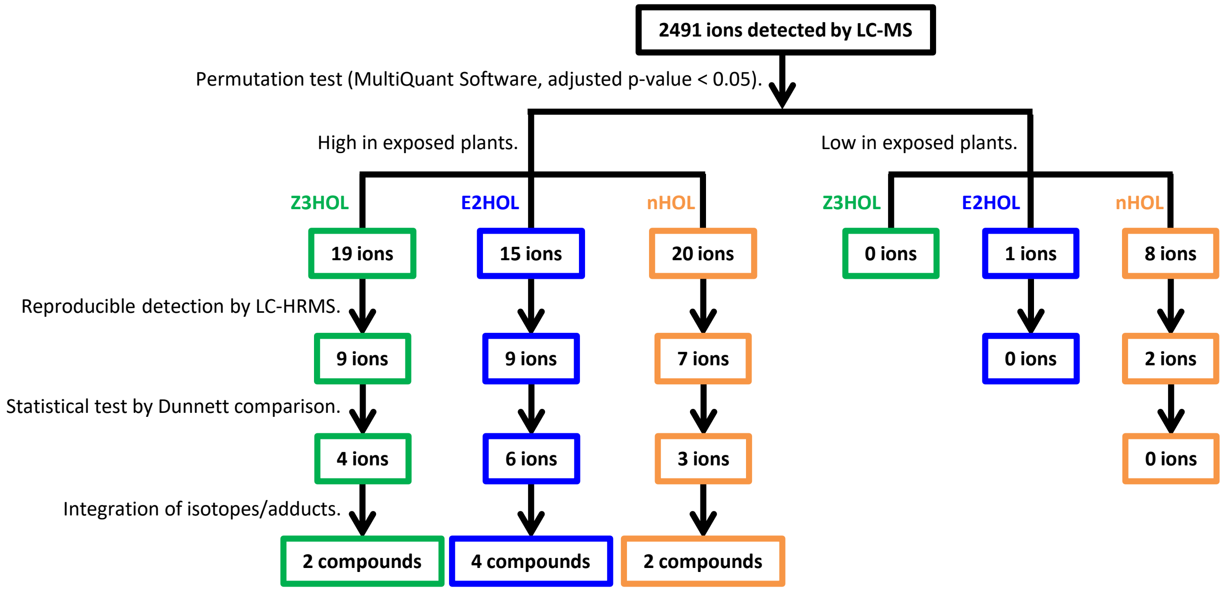

SUPPLEMENTARY FIGURE 1 | Work flow of metabolomic data analysis. Detailed descriptions are provided in the main text and the original data are provided as Supplementary Data.
