# Supplementary material for: Processing of Airborne Green Leaf Volatiles for Their Glycosylation in the Exposed Plants
Source: Front Plant Sci. 2021 Nov 16;12:721572. doi: 10.3389/fpls.2021.721572 (PMC8636985; doi:10.3389/fpls.2021.721572)
Supplement: Supplementary file 1 [file Data_Sheet_1.zip › SupplementaryMaterials/SupplementaryFigure2.pdf]

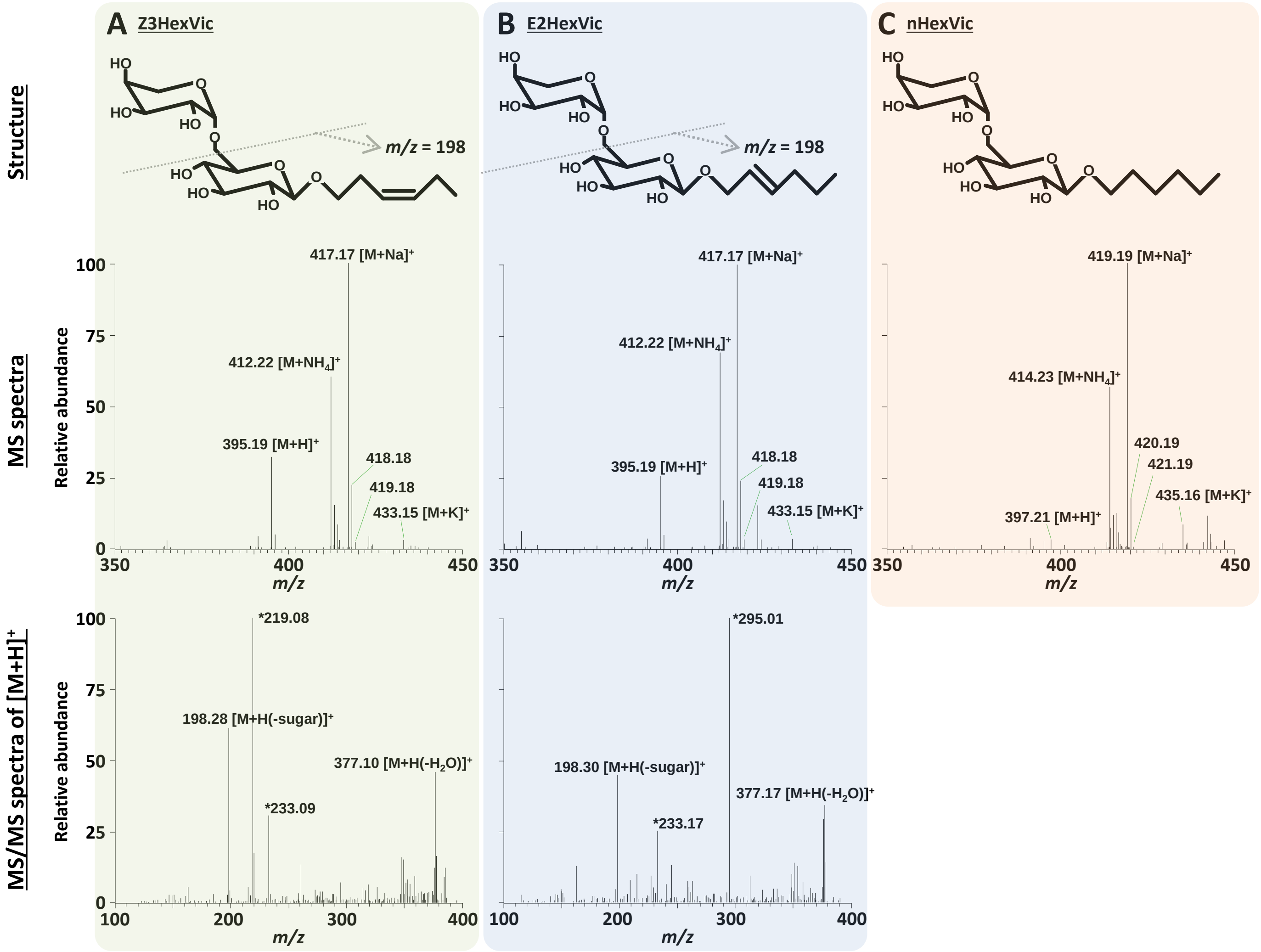

SUPPLEMENTARY FIGURE 2 | MS and MS/MS analyses of volatile glycosides. **(A)** Structure and MS and MS/MS spectra of Z3HexVic. Mass spectrum was derived from peak a of **Figure 3A**. **(B)** Structure and MS and MS/MS spectra of E2HexVic. Mass spectrum was derived from peak c of **Figure 3A**. **(C)** Structure and MS spectrum of nHexVic. Mass spectrum was derived from peak e of **Figure 3B**.
